# Characteristic contrast-enhanced MRI findings of nodular fasciitis and their chronological change

**DOI:** 10.1007/s11604-026-01962-2

**Published:** 2026-02-27

**Authors:** Teruko Ueno, Seiichi Matsumoto, Kyoko Yamashita, Keiko Hayakawa, Keisuke Ae, Masanori Saito, Satomi Kitai, Yoshinao Sato, Shohei Kiso, Kanae Takahashi, Takehiko Abe, Yui Tomita, Kensaku Mori

**Affiliations:** 1 Department of Diagnostic Radiology, Cancer Institute Hospital of Japanese Foundation for Cancer Research, 3-8-31 Ariake, Koto, Tokyo 135-8550 Japan; 2 Department of Orthopaedic surgery, Cancer Institute Hospital of Japanese Foundation for Cancer Research, Tokyo, Japan; 3 Department of Pathology, Cancer Institute Hospital of Japanese Foundation for Cancer Research, Tokyo, Japan; 4 Department of Radiology, National Cancer Center, Tokyo, Japan

**Keywords:** Nodular fasciitis, MRI, Contrast-enhanced imaging, Non-enhancing lesion, Soft tissue tumor, Chronological changes

## Abstract

**Purpose:**

This study aimed to identify characteristic contrast-enhanced MRI features of nodular fasciitis that aid in differentiating it from malignant tumors and to evaluate their chronological changes.

**Materials and methods:**

We analyzed 62 histologically confirmed nodular fasciitis lesions from 61 patients. All patients underwent T2-weighted and post-contrast MRI. Non-enhancing patterns were categorized as follows: type 1 (homogeneous enhancement), type 2 (central non-enhancement), type 3 (heterogeneous non-enhancement), and type 4 (predominantly non-enhancing). Type 4 was subdivided into type 4a (predominantly non-enhancing with minimal peripheral enhancement) and type 4b (completely non-enhancing with perilesional enhancement). MRI findings from 50 malignant tumors served as controls.

**Results:**

The initial MRI patterns included type 1 (*n* = 24, 39%), type 2 (*n* = 17, 27%), type 3 (*n* = 9, 15%), and type 4 (*n* = 12, 19%). Among the 12 type 4 lesions, 8 exhibited type 4b. Three additional lesions evolved into type 4b, totaling 11 (17%) of the 62. A follow-up MRI was performed in 5 type 4b lesions; 4 demonstrated changes to other types, although without a consistent transformation pattern. Type 4b lesions were absent in the malignant control group.

**Conclusion:**

In this cohort, type 4b was observed in nodular fasciitis and not in the malignant controls evaluated; specificity beyond this control spectrum remains unknown and requires external validation. The temporal evolution from type 4b to other patterns was variable, potentially explaining the previously reported heterogeneity in MRI findings. Follow-up imaging may help explain the variability in reported MRI appearances.

## Introduction

Nodular fasciitis is a proliferative lesion composed of fibroblasts and myofibroblasts, forming a discrete or infiltrative mass. It primarily affects the subcutaneous tissue, fascia, and muscle of individuals under 50 years of age and often enlarges rapidly, with associated tenderness and pain. Clinically, it is frequently mistaken for a malignant tumor, although spontaneous regression is well recognized. The most common locations are the upper extremities, followed by the trunk, head and neck, and lower extremities. In 71% of cases, the lesion measures less than 2 cm in diameter and rarely exceeds 4 cm [1].

Although long considered a reactive proliferative process, chromosomal translocation resulting in USP6 overexpression has led to its reclassification as a self-limited neoplastic lesion [2, 3]. As the lesion tends to regress spontaneously, resection is unnecessary when the diagnosis is definitive. However, because its early clinical presentation often mimics malignancy, and distinguishing it from low-grade sarcoma using small-needle biopsy specimens can be challenging [4–6], recognizing its imaging features is crucial for supporting a histologic diagnosis.

According to previous reports on the imaging features of nodular fasciitis, T2-weighted images frequently demonstrate a variety of nonspecific signal patterns [7, 8], and the lesion may show an infiltrative growth pattern mimicking malignancy [8–10]. In other words, there are no pathognomonic findings on non-contrast MRI.

On contrast-enhanced MRI, both diffuse enhancement and central non-enhancing regions, thought to represent cystic changes or mucin retention, have been described [5, 7, 11]. However, these features are not specific and can also be observed in malignant tumors.

Typically, non-enhancing regions on MRI correspond to areas of tumor necrosis or degeneration [12–14], a phenomenon observed across both benign and malignant neoplasms. However, given the self-limited biology of nodular fasciitis and its known potential for spontaneous regression [15], non-enhancing regions in this condition may reflect a different pathophysiological process than those in malignancy. Indeed, previous studies have reported a characteristic enhancement pattern consisting of a central non-enhancing area with peripheral enhancement [16, 17].

Additionally, molecular testing for USP6 gene fusion has enabled more definitive diagnosis from limited biopsy material, contributing to an increase in cases managed with surveillance alone. This shift has resulted in more instances in which serial MRI is performed to monitor temporal evolution. Previous studies have noted that MRI findings in nodular fasciitis change over time [18, 19]. Furthermore, USP6 split-signal positivity is reportedly more frequent in younger lesions, whereas older lesions show collagen-dense stroma [15], suggesting histologic changes related to senescence. Therefore, corresponding chronological changes in MRI findings are expected.

To date, few studies have evaluated the temporal evolution of contrast-enhanced MRI findings in large case series. Thus, we retrospectively analyzed nodular fasciitis cases using contrast-enhanced MRI, emphasizing the morphology of the non-enhancing component. In addition, Wang et al. observed that the previously described pattern, non-enhancement of the tumor with perilesional tissue enhancement, can appear during the disease course [7]. We examined how this feature changes over time.

## Materials and methods

This retrospective study was approved by the Institutional Review Board of the Cancer Institute Hospital of the Japanese Foundation for Cancer Research (Approval Number: 2023-GB-193; approved on May 1, 2024). The requirement for informed consent was waived because of the retrospective study design. Of the 71 cases histopathologically diagnosed with nodular fasciitis and treated at our hospital between January 2006 and August 2021, 61 were selected in which both T2-weighted imaging and contrast-enhanced MRI were performed in at least two planes. Of these 61 cases, 60 involved single lesions; however, one patient had two distinct lesions on the thigh [18], resulting in a total of 62 lesions.

**Fig. 1:**
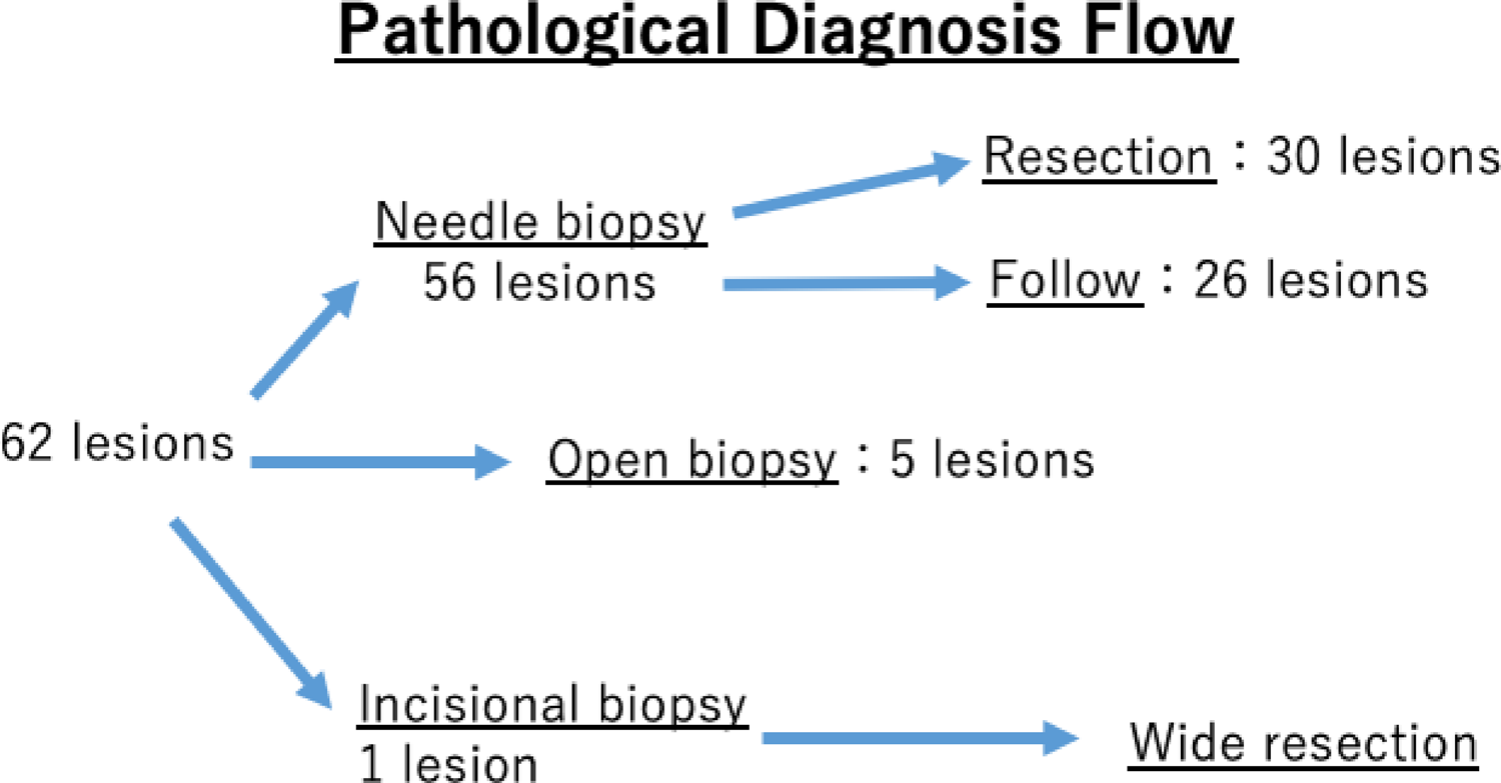
Pathological diagnostic flow. The diagram illustrates the pathological diagnostic approach for nodular fasciitis. While most cases were managed through resection or follow-up based on needle biopsy results, excisional or incisional biopsy was preferred when needle biopsy proves challenging due to factors such as tumor location or diagnostic uncertainty

The histological diagnostic methods were categorized as follows (Fig. 1): 30 of the 62 lesions were diagnosed solely by needle biopsy, whereas 26 underwent surgical resection because needle biopsy did not confirm nodular fasciitis. Five lesions underwent excisional biopsy rather than needle biopsy because of their proximity to vessels or nerves or because of their small size. One case involved material from an excisional biopsy performed at a referring institution in which malignancy could not be excluded; wide excision was subsequently performed. In 2019, our institution began testing for USP6 gene rearrangement using FISH analysis of needle biopsy specimens.

**Fig. 2:**
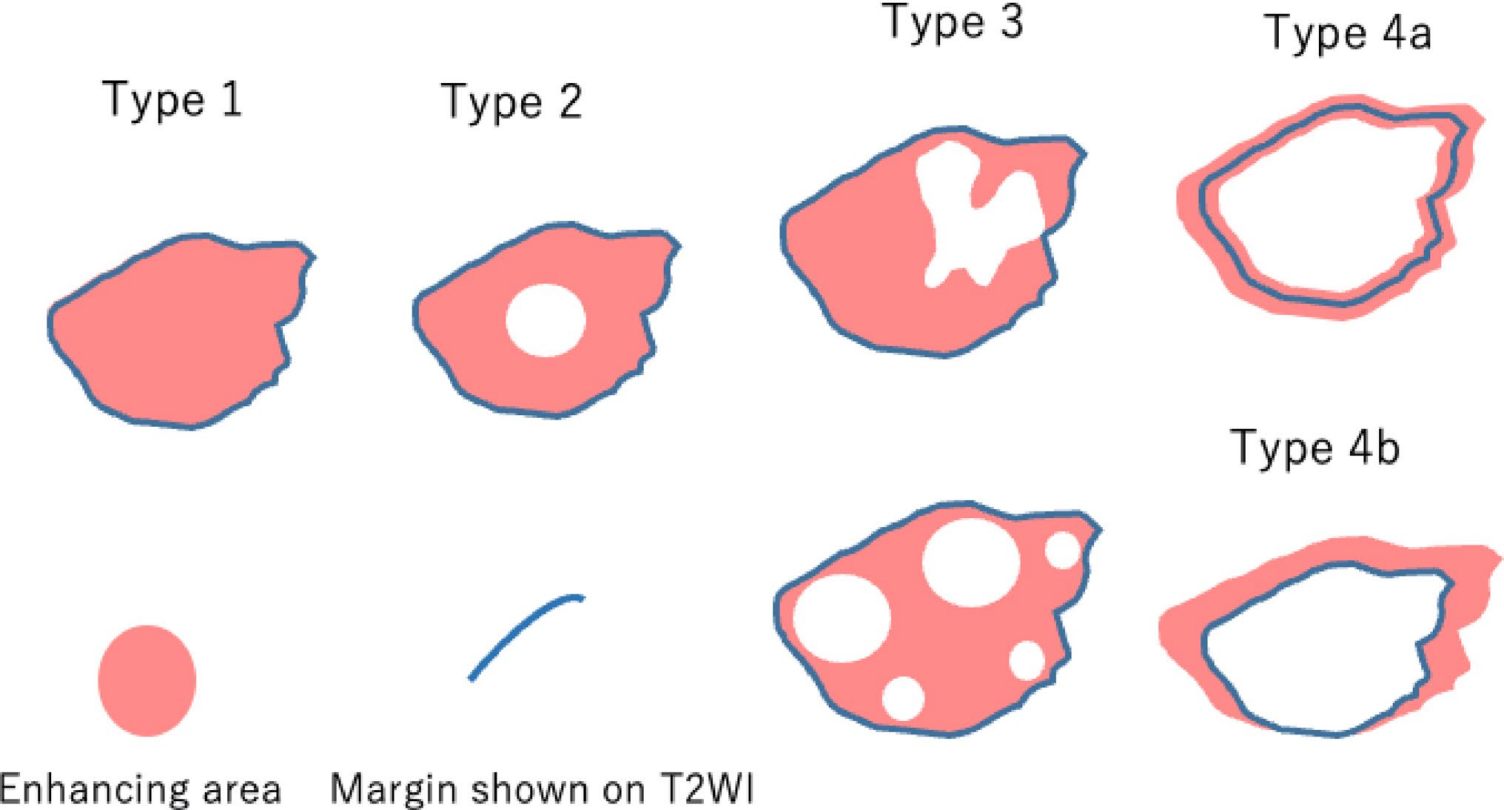
Classification of non-enhancing area. The schematic diagram shows the classification of the distribution of non-enhancing area: the tumor margins indicated by abnormal signal in T2-weighted images are indicated by blue lines, enhancing areas are indicated by red, and non-enhancing area indicated by white. Type 1: The tumor shows homogeneous enhancement throughout. Type 2: A non-enhancing area is present only at the center of the tumor. Type 3: The tumor contains heterogeneous non-enhancing regions that do not meet the criteria for type 2 or type 4. Type 4: The tumor is predominantly non-enhancing. Type 4a: Predominantly non-enhancing with minimal peripheral enhancement. Type 4b: Completely non-enhancing with perilesional enhancement

**Fig. 3:**
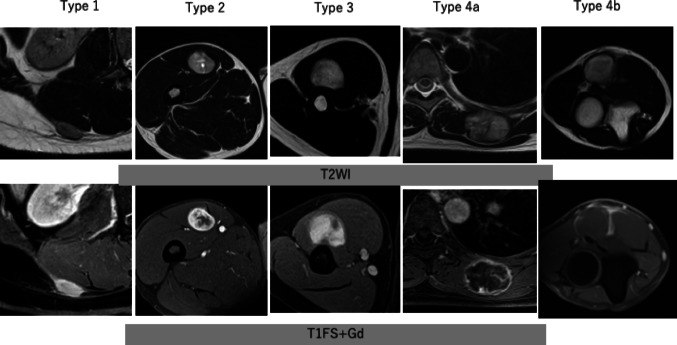
MRI images of a case reflecting the four type classifications. The upper panel is the T2-weighted axial image, and the lower panel is a post-contrast fat suppressed T1-weighted axial image. Type 1 shows entire tumor enhancement ; type 2 shows non-enhancing area only in the center; type 3 shows non-enhancing area including outside the center; and type 4a shows a predominantly non-enhancing area with minimal peripheral enhancement. Type 4b shows a completely non-enhancing area with perilesional enhancement

The correlation between tumor extent on T2-weighted imaging and the morphology of non-enhancing regions was classified into four types (Figs. 2, 3).

Type 1: The tumor shows homogeneous enhancement throughout.

Type 2: A non-enhancing area is present only at the center of the tumor.

Type 3: The tumor contains heterogeneous non-enhancing regions that do not meet the criteria for type 2 or type 4.

Type 4: The tumor is predominantly non-enhancing.

Type 4a: Predominantly non-enhancing with minimal peripheral enhancement.

Type 4b: Completely non-enhancing with perilesional enhancement.

We defined peripheral enhancement as contrast enhancement involving the lesion margin and its adjacent inner portion within the area identified as hyperintense or hypointense on T2-weighted images, whereas perilesional enhancement was defined as enhancement extending beyond the outer border of the T2-abnormal area. Although the disease entity and underlying mechanisms differ, we adopted these definitions because their morphological features are similar to those described by Yu et al. and Hu et al. for hepatic metastases [20].

Additionally, because this lesion has been reported to occur near the fascia [8, 21], we also examined the spatial relationship between the tumor and adjacent fascia on type 4b MR images.

Primary malignant soft-tissue tumors diagnosed between November 2005 and December 2018 were selected as the control group. Inclusion criteria were as follows: (1) longest diameter of 5 cm or less; (2) initial presentation with pre-treatment contrast-enhanced MRI; and (3) fibrous tumors that histopathologically required differentiation from nodular fasciitis. This resulted in 50 cases. The cohort included 32 undifferentiated pleomorphic sarcomas (UPS), 8 solitary fibrous tumors (SFT), 6 myxofibrosarcomas, and 2 each of fibrosarcoma and low-grade fibromyxoid sarcoma. These tumors were similarly classified into four enhancement-based types. Image interpretation was performed by consensus between two specialists, a radiologist and an orthopedic surgeon, both experienced in musculoskeletal tumor imaging and management.

### Statistical analysis

The distribution of type classifications between nodular fasciitis and malignant controls was compared using the chi-square test without continuity correction. Adjusted residuals were calculated to identify categories contributing to the overall association. A two-sided *p* < 0.05 was considered statistically significant, whereas a value of 0.05 ≤ *p* < 0.10 was interpreted as indicating a trend toward significance. All statistical analyses were performed using R software (version 4.5.1; R Foundation for Statistical Computing, Vienna, Austria).

## Results

The longest tumor diameter ranged from 5 to 64 mm, with a median of 21.5 mm and a mean of 25.0 mm. Notably, none of the patients demonstrated re-enlargement of the lesion after an initial reduction in size. Patient age ranged from 8 to 85 years (mean, 40.0 years) and included 31 men (32 lesions) and 30 women. Lesions were located in the upper extremity and shoulder girdle (*n* = 28), trunk (*n* = 10), and lower extremity (*n* = 22), with 25 lesions arising in the subcutaneous tissue and 37 in the deep soft tissue.

### Number of MRI examinations and initial MRI findings

A total of 118 MRI examinations were performed for 62 lesions. The distribution was as follows: 39 lesions underwent a single MRI, 11 had two MRIs, 4 had three MRIs, and 8 lesions underwent more than four examinations. Among the 62 lesions, 23 (37%) underwent more than two MRI examinations during follow-up. After the introduction of USP6 rearrangement testing, observational management became more feasible, resulting in a trend toward increased use of serial MRI.

The non-enhancing patterns on initial contrast-enhanced MRI were distributed as follows: type 1, 24 lesions (39%); type 2, 17 (27%); type 3, 9 (15%); and type 4, 12 (19%). Type 1, defined by uniform enhancement of the lesion, was the most frequently observed pattern.

### Chronological change of type 4b

**Fig. 4:**
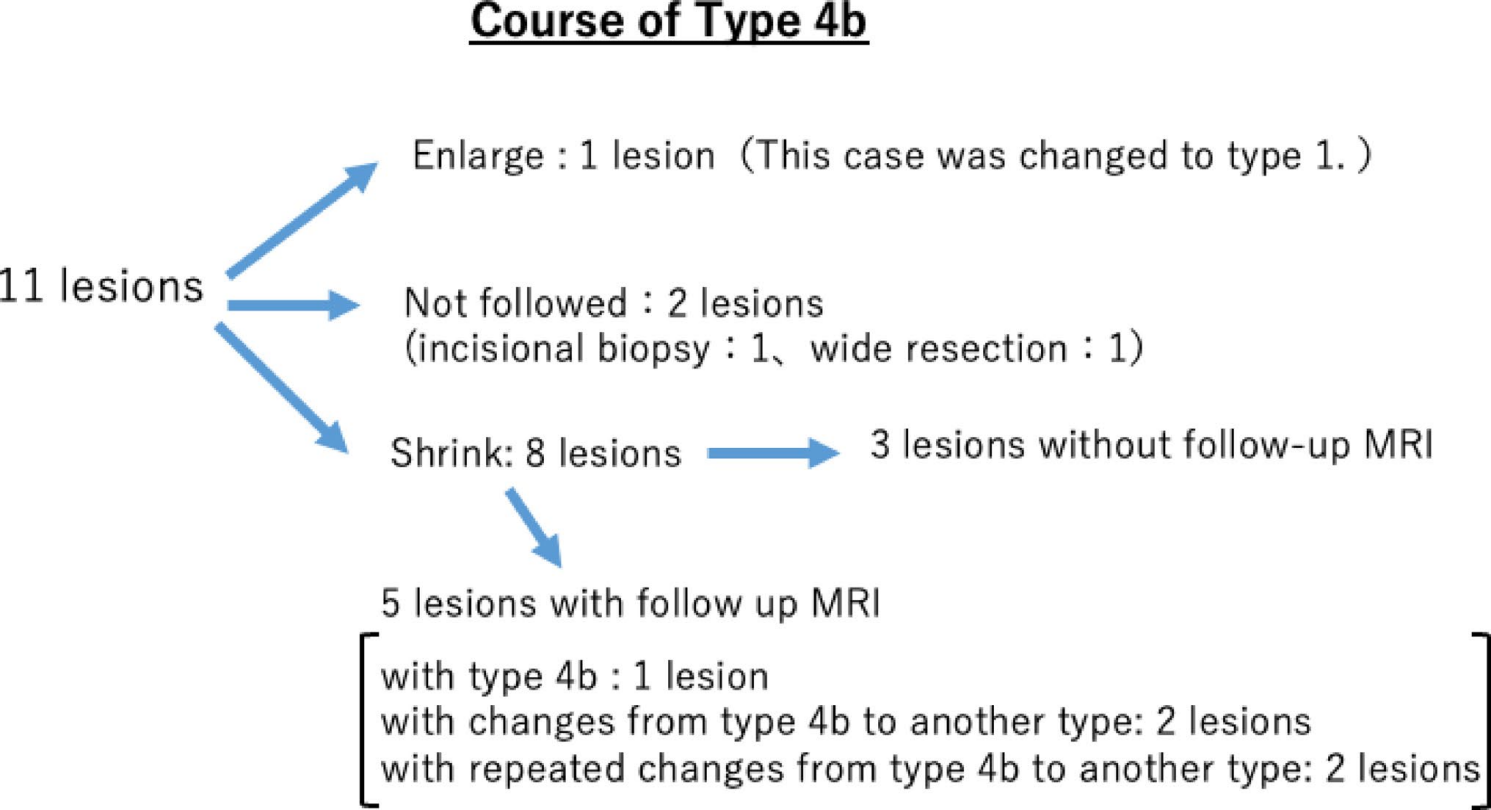
Clinical course of type 4b. This chart illustrates the clinical course of 11 lesions initially presenting as type 4b. Among these lesions: One lesion was excised due to tumor growth occurring simultaneously with the change from type 4b to type 1. Two lesions were resected after demonstrating type 4b. The remaining eight lesions showed a reduction in size after presenting as type 4b. Of these eight lesions: Three of them no longer underwent MRI evaluation. Five lesions demonstrated type 4b on follow-up MRI. In these five lesions: One lesion exhibited shrinkage while maintaining the type 4b pattern. In the remaining four lesions, the type 4b pattern transitioned to other types

**Fig. 5:**
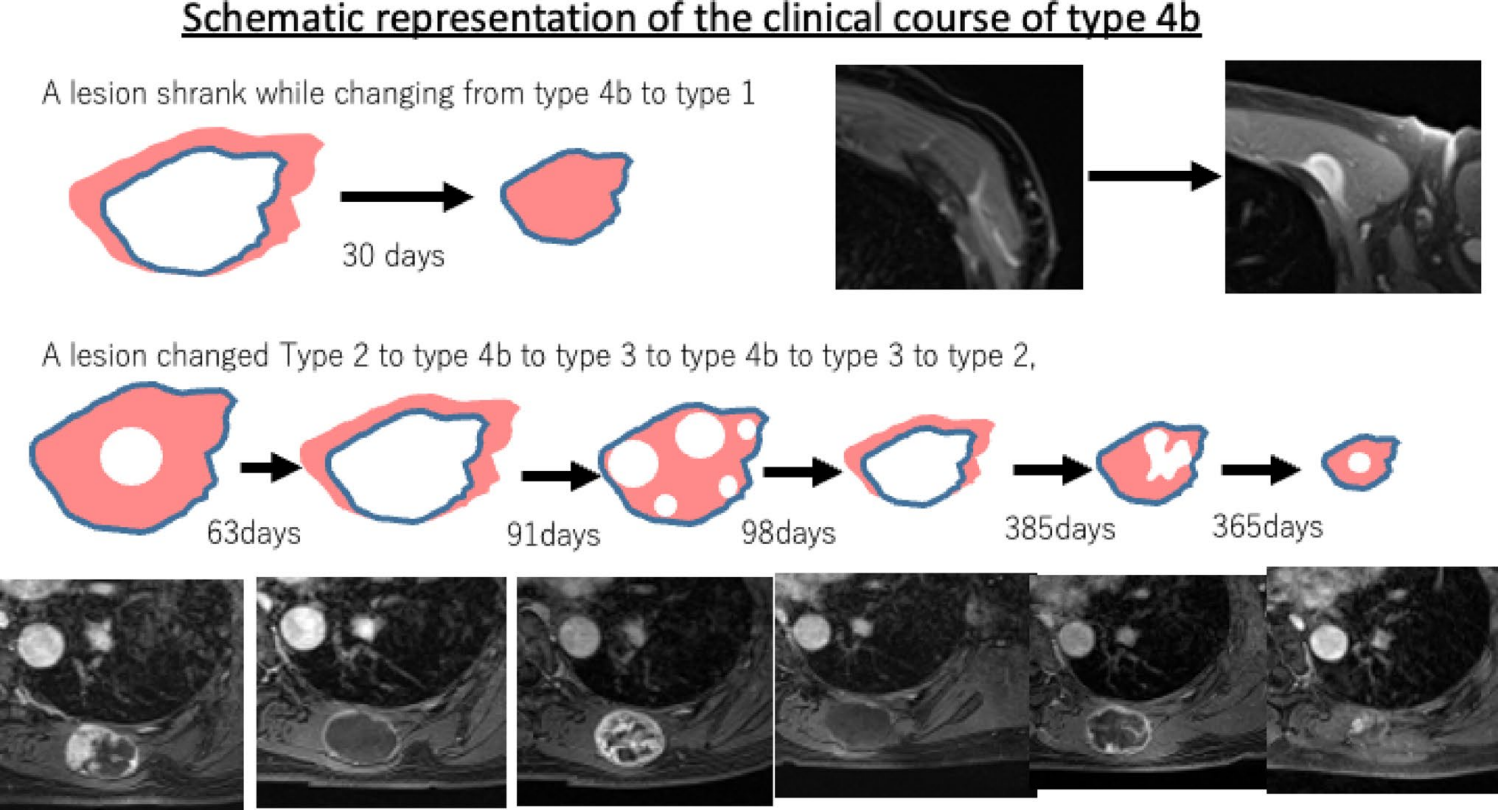
Schematic representation of the clinical course of type 4b. The schematics illustrate the chronological changes observed in five cases of type 4b that were monitored by MRI. It was evident that previously non-enhancing areas could exhibit re-enhancement and that the patterns of change varied across different types, without consistent patterns

On initial MRI, 8 cases were classified as type 4b. Additionally, 3 cases initially assigned to other types transitioned to type 4b during the disease course. Considering both initial and follow-up assessments, type 4b was observed in 11 of 62 lesions (17%). The follow-up findings for these 11 lesions after confirmation of type 4b are presented in Figs. 4 and 5. In one case, the lesion transitioned from type 4b to type 1 with an increase in size and was subsequently resected. Additionally, one lesion that was technically difficult to biopsy and another with indeterminate biopsy findings were both resected after being categorized as type 4b, leaving no follow-up MRI data. The remaining 8 lesions demonstrated type 4b and subsequently showed regression. Type 4b was observed 18 times across these cases, including 7 examinations performed before biopsy and 10 after biopsy. The interval between biopsy and the appearance of type 4b findings ranged from 5 to 258 days (median, 70 days; mean, 99.1 days). Type 4b enhancement is not a transient change limited to the immediate post-biopsy period and is unlikely to be consistently induced by biopsy.

In three of these cases, shrinkage was confirmed by ultrasound and palpation, and MRI was not repeated. In the remaining five lesions, MRI was performed after type 4b classification. These five cases had a wide range of observation periods, from 38 to 1,548 days (median, 281 days; mean, 484 days), indicating that both short- and long-term follow-up cases were included. One lesion retained the type 4b pattern while shrinking. Two lesions regressed while transitioning to type 1 or type 3. The remaining two demonstrated fluctuating enhancement types during regression. In one lesion, the type shifted from type 4b to type 2 to type 4b to type 2 to type 4b to type 2. In the other, the sequence was type 2 to type 4b to type 3 to type 4b to type 3 to type 2. These observations suggest that the non-enhancing region may re-enhance during disease evolution and that type 4b may represent a transient phase in the natural course of nodular fasciitis.

**Fig. 6:**
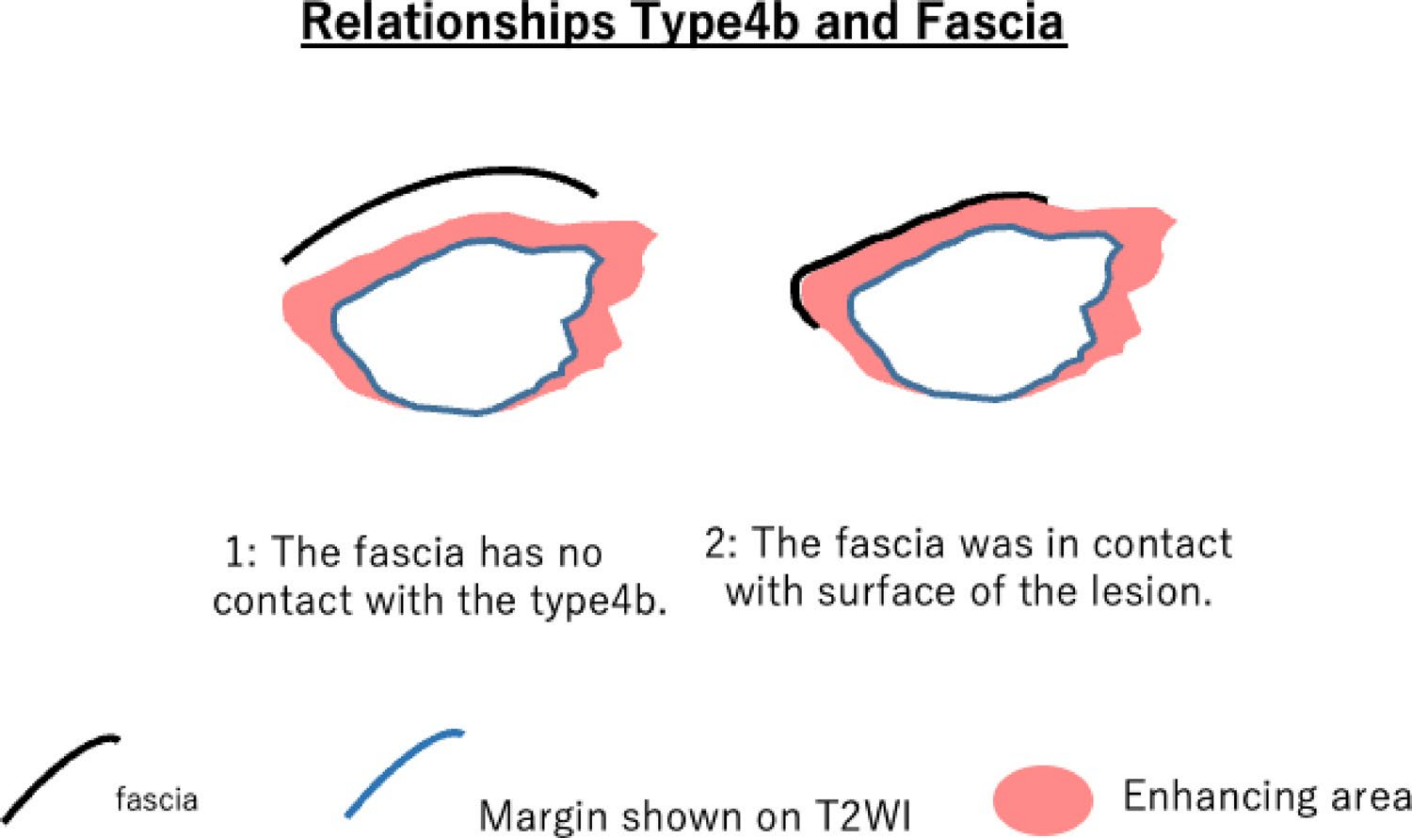
Relationships between type 4b and fascia. Schematic illustration showing the relationship between the enhancing area and the fascia (black line) in type 4b lesions. (1) The enhancing area shows no contact with the fascia. (2) The enhancing area is in contact with the fascia along the lesion surface

Among the 11 lesions demonstrating type 4b, the relationship between the enhancing peritumoral tissue and fascia was assessed. In nine lesions, fascial contact involved less than half of the tumor circumference. In two lesions, the contact extended over more than half. In one of the nine lesions, the enhancing area showed no fascial contact (Fig. 6). Thus, although peritumoral enhancement in type 4b was associated with the fascia in some cases, this finding was inconsistent across lesions.

## Histopathological findings of type 4b

**Fig. 7:**
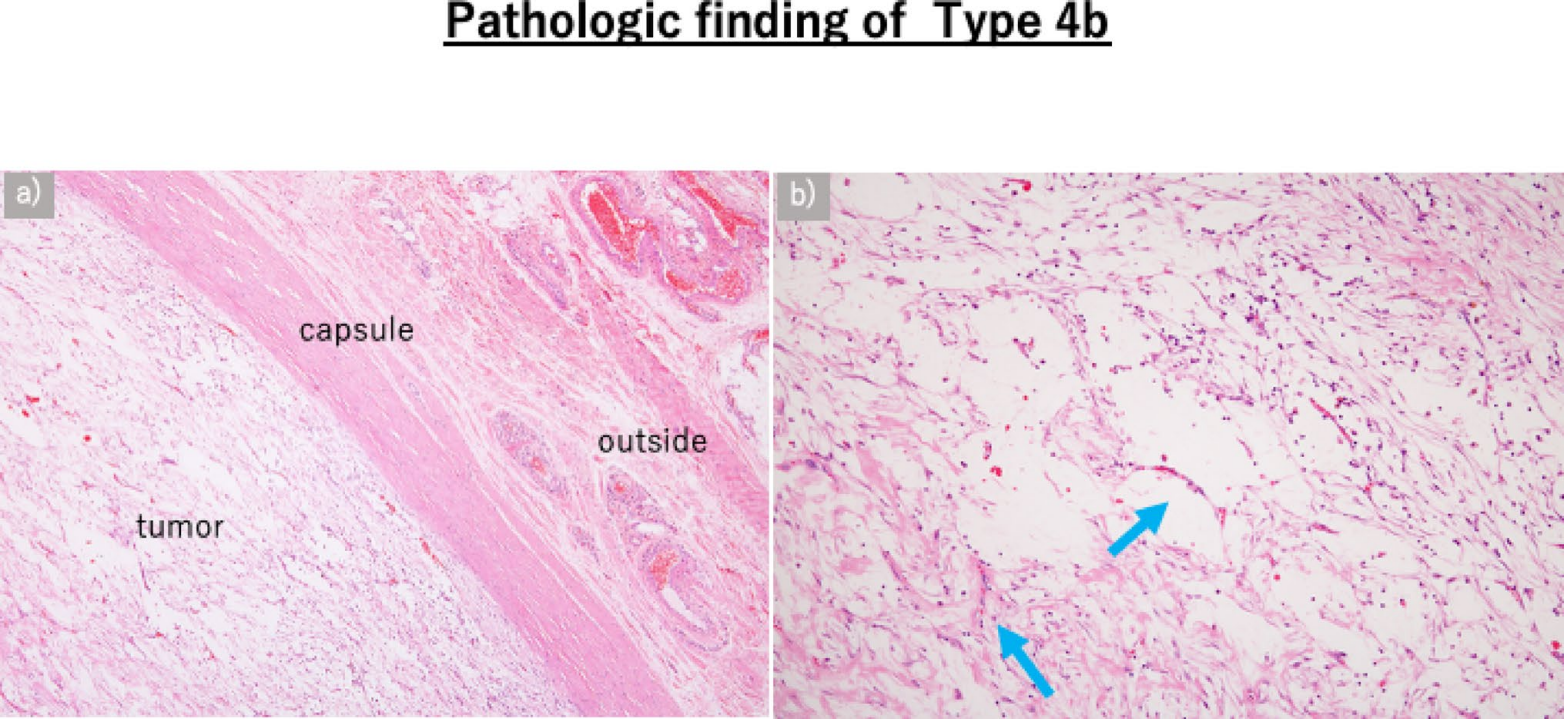
Histopathologic findings of the wide resected type 4b case. **a** Outside the capsule, there was an increase of arteries and veins with normal morphology without neoplastic lesions. **b** Inside the tumor, the overall cell density was low, suggesting cell depletion due to apoptosis. The tumor was characterized by sparse fibrosis and diffusely distributed capillaries (→). No necrosis was observed

Although based on a single case, wide resection permitted histological evaluation of both the tumor and the peritumoral tissue characteristic of type 4b (Fig. 3). This revealed a well-defined fibrous capsule that nearly completely encased the tumor margin (Fig. 7). Transitional zones between the tumor and capsule were also noted. Outside the capsule, no definite tumor components were identified, but increased vascular and venous density was observed. The peritumoral contrast enhancement seen on MRI was believed to reflect this vascular proliferation (Fig. 7a). Within the capsule, the tumor showed no necrosis. However, overall cellularity was low, with a pronounced decline in cell density toward the central portion. Additionally, sparse fibrotic zones and diffusely distributed capillaries were present within the lesion. These capillaries were presumed to contribute minimally to the enhancement observed on MRI.

### Control group

**Fig. 8:**
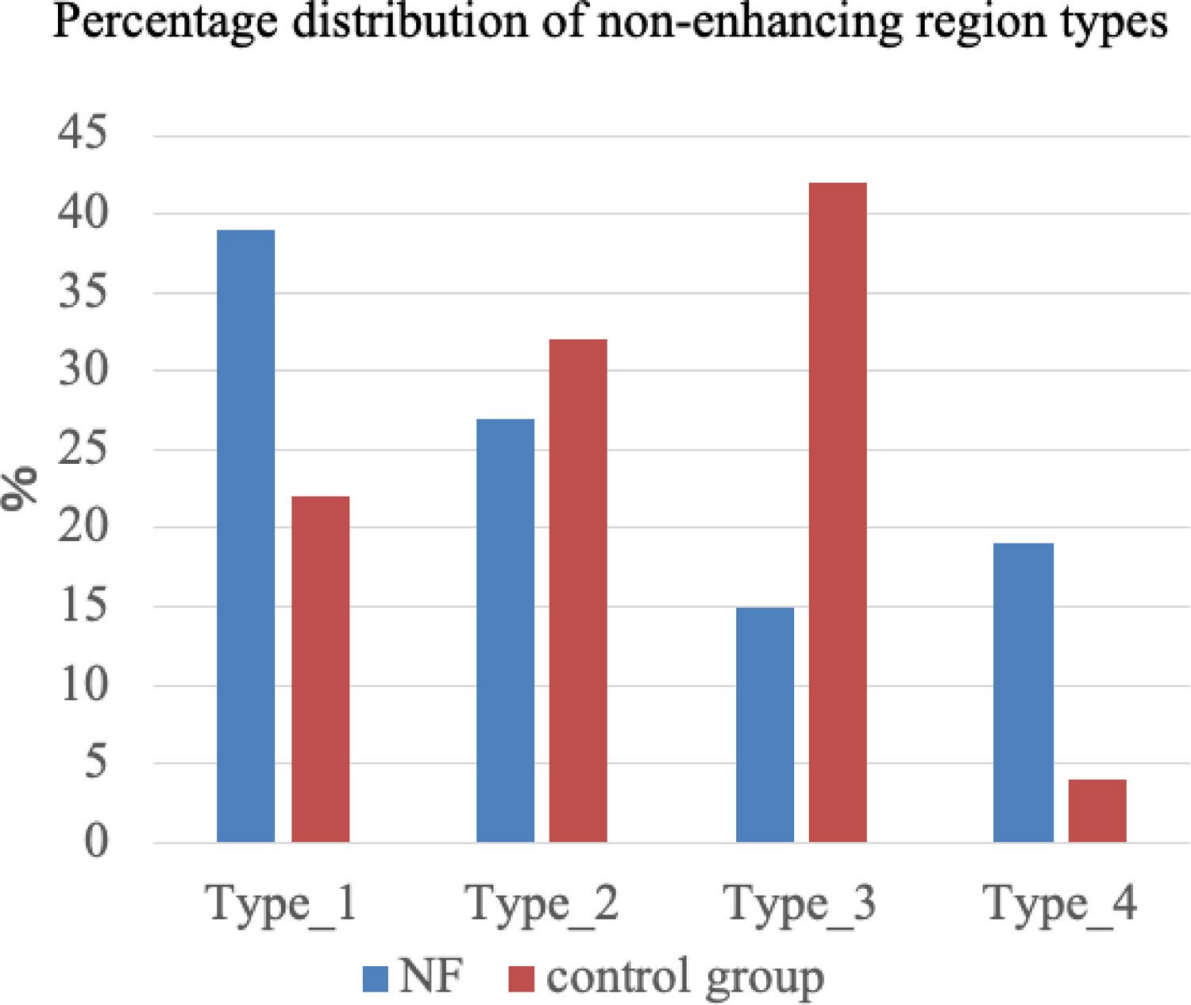
Percentage distribution of non-enhancing region patterns. The bar chart illustrates the percentage distribution of non-enhancing region patterns (Types 1–4) in nodular fasciitis (NF; blue bars) and the malignant control group (red bars). NF shows a higher proportion of Types 1 and 4, whereas the malignant control group predominantly exhibits Type 3

**Fig. 9:**
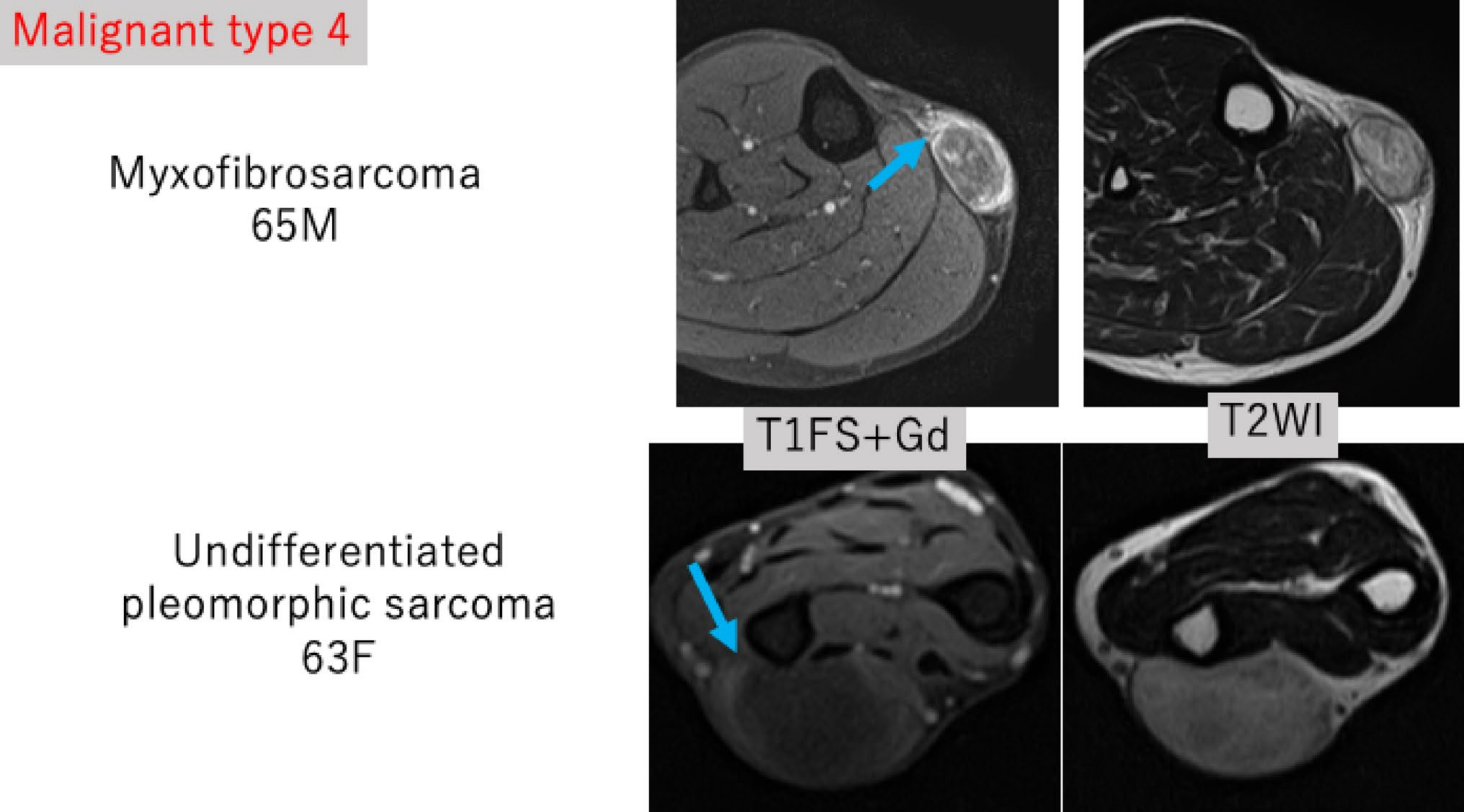
Type 4a enhancement pattern in malignant soft-tissue tumors. Upper row: myxofibrosarcoma. Lower row: undifferentiated pleomorphic sarcoma. In both cases, limited enhancement was observed within the peripheral region of the lesion. Enhancement extended from the tumor periphery into the perilesional tissue, while most of the tumor parenchyma remained non-enhancing. This enhancement pattern is distinct from type 4b

The mean size of the 50 malignant tumors in the control group was 39 mm. The cohort comprised 27 women and 23 men, aged 13–84 years (mean, 59.8 years; median, 63 years). Tumor sites included the upper extremity and shoulder girdle (*n* = 17), trunk (*n* = 14), and lower extremity (*n* = 19). Thirty lesions arose in the subcutaneous tissue and 20 in the deep soft tissue. The non-enhancing patterns were categorized as follows: type 1 in 11 cases (22%), type 2 in 16 cases (32%), type 3 in 21 cases (42%), and type 4 in 2 cases (4%). Cases with uniform contrast enhancement throughout the lesion (type 1) and those with non-enhancement across the entire lesion (type 4) were less frequent compared with nodular fasciitis (Fig. 8). Notably, type 3, which was the least common pattern in nodular fasciitis, was the most prevalent in the control group. The histologic subtypes of the two type 4 tumors were myxofibrosarcoma and UPS. On contrast-enhanced MRI, enhancement was observed extending from the tumor periphery into the perilesional tissue, while the majority of the tumor parenchyma remained non-enhancing (Fig. 9). This enhancement pattern is distinct from type 4b, in which the tumor, including its peripheral margin, shows no internal enhancement and enhancement is confined exclusively to the perilesional tissue along the tumor circumference.

## Comparison of type classification

A chi-square test revealed a significant association between diagnosis category and type classification, with a moderate effect size (Cramer’s V = 0.374) (Table 1). Residual analysis demonstrated that type 3 was significantly more frequent in the malignant control group (adjusted residual = 3.265, *p* < 0.01), whereas type 4 was significantly more frequent in the NF group (adjusted residual = 2.443, *p* < 0.05). Type 1 showed a trend toward a higher frequency in the NF group (adjusted residual = 1.897, 0.05 ≤ *p* < 0.10). No significant difference was observed for type 2.

**Table 1:** Comparison of enhancement type distribution between nodular fasciitis and the malignant control group

	Type 1	Type 2	Type 3	Type 4
NF (*n* = 62)	24 (39%) ▲ † (AR = 1.897)	17 (27%) (AR = 0.529)	9 (15%) ▽ ** (AR = − 3.265)	12 (19%)▲ * (AR = 2.443)
Control (*n* = 50)	11 (22%) ▽ † (AR = − 1.897)	16 (32%) (AR = 0.529)	21 (42%)▲ (AR = 3.265)	2 (4%)▽ * (AR = − 2.443)

AR, adjusted residual; *p* < 0.05; *p* < 0.01; †0.05 ≤ *p* < 0.10 (trend); ▲ observed > expected; ▽ observed < expected

• Overall association: χ²(3) = 15.696, *p* = 0.001; Cramer’s V = 0.374

## Discussion

Recently, the identification of fusion genes has enabled the accurate diagnosis of nodular fasciitis using small biopsy specimens. Consequently, as observed in our cohort, there has been an increase in cases managed through observation until tumor regression occurs following biopsy. A decrease in signal intensity on T2-weighted images over time has been previously reported [18, 19]. This change is believed to reflect a reduction in mucus and blood vessels, along with progressive accumulation of fibrous tissue [15, 22]. Shrinkage of the enhancing area over time has also been described by Sukpanichyingyong et al. [19]. These shrinkage features are clinically relevant because they support a benign process such as nodular fasciitis. However, such features can only be assessed through longitudinal observation. Therefore, progressive reduction in size and T2 signal intensity has limited clinical utility in differentiating benign from malignant tumors. Additionally, findings on T2-weighted imaging are highly variable and nonspecific [23], complicating reliable distinction between benign and malignant lesions.

Wang et al. reported contrast enhancement of the tumor periphery with a non-enhancing central region in nodular fasciitis, termed the “inverted target sign” [7]. This was attributed to peripheral hypervascularity and central cystic change. A histologic tendency for cyst formation in the central region of nodular fasciitis has also been described [3, 7, 8]. In our series, lesions were classified as either type 2 (central non-enhancement) or type 4 (predominantly non-enhancing), with type 2 occurring more frequently. Additionally, some lesions exhibited dynamic transitions between types during follow-up. Wang et al. [7] also reported that the enhancing portion of the “inverted target sign,” which corresponds to type 2 in our classification, gradually diminished over time, evolving into a pattern with peripheral-only enhancement. Although precise classification based solely on their data is difficult, the post-transition patterns appear to correspond to type 4a or type 4b. These imaging changes are consistent with our findings.

In summary, the reported MRI features of nodular fasciitis likely represent temporal phases in a dynamic biological process. This evolution may explain the spectrum of imaging findings described in previous reports.

The known histologic changes in nodular fasciitis over time, namely “progressive fibrosis and central cyst formation” [3, 6, 8], are considered primary contributors to the development of types 2 and 4. In our cases, we demonstrated that lesions can transform not only into types 2 and 4 but also into other types. For example, two lesions decreased in size while transitioning from type 4b to type 1 or type 3, suggesting that, in addition to fibrosis and cystic change, other histopathological processes may occur over time. Wang et al. further clarified that enhancing areas correspond to regions containing myxoid components, whereas non-enhancing areas reflect cystic cavities. The observed MRI findings are attributed to this heterogeneous tissue distribution [7]. These longitudinal changes in contrast-enhanced MRI features may reflect evolving distributions of mucinous and cystic content. Wang et al. also reported that central non-enhancing tissue often contains “more extracellular matrix, some fluid-filled spaces,” which differs from the histologic findings in our type 4b case. Taken together, these observations suggest that multiple factors likely contribute to the development of non-enhancing regions.

Among the 11 cases classified as type 4b, one exhibited size increase, nine showed regression, and one was resected, precluding clinical follow-up. These findings suggest that type 4b is strongly associated with a tendency toward tumor shrinkage. Histological evaluation of the resected type 4b specimen showed no necrosis. Although the tumor center exhibited low vascularity, a high density of vessels was observed outside the capsule. This vascular pattern likely contributes to the characteristic imaging features. Observed transitions from type 4 to type 2 or type 1 indicate that previously non-enhancing areas became re-enhancing over time. This supports the hypothesis that non-enhancing areas, defined by low cellularity and vascularity, may subsequently regain both. These findings suggest that nodular fasciitis may enter a phase in which apoptotic tumor cells [15, 24] coexist with proliferating cells, and vascular supply undergoes dynamic changes. This biologic interplay may account for the inconsistent patterns of contrast enhancement on MRI. If enhancement characteristics fluctuate over time, imaging findings will vary depending on scan timing, contributing to the wide spectrum of reported MRI features. Previous reports have highlighted the variability in T2-weighted and contrast-enhanced MRI findings of nodular fasciitis [8, 23, 25]. Our results offer a plausible explanation for this variability.

Wu et al. reported the presence of inverted target signs in 11% of cases [17]. Their study suggests that nodular fasciitis exhibits inverted target signs somewhat more frequently than other soft-tissue lesions. They also described one case (3.7%) with a finding resembling type 4b, termed the “solar halo sign,” considered a notable feature not typically observed in malignant tumors. In our study, type 4b was identified in 17% of cases. If this corresponds to the “solar halo sign” described by Wu et al., the frequency in our cohort is substantially higher. Because our study incorporated an analysis of temporal evolution, the increased detection of type 4b may be partly attributable to this longitudinal imaging approach. Currently, we cannot propose an alternative explanation. However, the higher frequency and dynamic nature of this finding, previously underemphasized, warrant further attention.

The chi-square test revealed that type 3 was significantly more frequent in malignant control group, whereas type 4 was significantly more frequent in nodular fasciitis. Type 1 also tended to be more prevalent in nodular fasciitis. These results imply that nodular fasciitis lesions tend to exhibit homogeneous enhancement or predominantly non-enhancing areas, suggesting relatively homogeneous tissue composition throughout their course. In contrast, malignant tumors were characterized by a higher frequency of type 3, reflecting their inherent internal heterogeneity, as previously recognized. Thus, the non-enhancing pattern classification likely reflects the distinct biological characteristics of nodular fasciitis and our malignant control group.

Type 4b was observed exclusively in nodular fasciitis and not in our malignant group. The temporal evolution of this pattern was inconsistent, with diverse imaging changes noted. In some cases, the type 4b pattern persisted as the lesion regressed, whereas in others, previously non-enhancing regions became enhanced. These findings are consistent with prior reports describing the heterogeneous MRI appearances of nodular fasciitis.

### Limitations

This study has several limitations. First, because it was retrospective and spanned a long period (2006–2021), the imaging protocols were heterogeneous. The contrast agent dose, injection rate, and administration method (manual versus power injector) were not standardized, and MRI examinations were performed on multiple scanners using different fat-suppression techniques. Although these factors introduced variability, the primary endpoint—a qualitative assessment of equilibrium-phase enhancement patterns—was considered minimally affected by such protocol differences.

Additionally, in current practice, the number of cases managed conservatively has increased, likely due to USP6 detection in needle biopsy specimens. Inter-reader reproducibility was not assessed because image interpretation was performed by consensus.

Type 4b patterns were not identified in our malignant control group and may be useful in differentiating nodular fasciitis from malignancy. However, the control group in this study was limited to pathologically confirmed tumors requiring histologic differentiation within a restricted size range for evaluating non-enhancing areas. Therefore, the control group may not represent the overall characteristics of malignant tumors.

## Data Availability

The datasets generated and/or analysed during the current study are not publicly available due to the inclusion of personal and clinical information of the participants.

## References

[1] Bernstein KE, Lattes R. Nodular (pseudosarcomatous) fasciitis, a nonrecurrent lesion: clinicopathologic study of 134 cases. Cancer. 1982;49(8):1668–78. 6279273 10.1002/1097-0142(19820415)49:8<1668::aid-cncr2820490823>3.0.co;2-9

[2] Erickson-Johnson MR, Chou MM, Evers BR, Roth CW, Seys AR, Jin L, et al. Nodular fasciitis: a novel model of transient neoplasia induced by MYH9-USP6 gene fusion. Lab Invest. 2011;91(10):1427–33. 21826056 10.1038/labinvest.2011.118

[3] WHO Classification of Tumours Editorial Board. Soft Tissue and Bone Tumours. Lyon (France): International Agency for Research on Cancer; 2020. (WHO classification of tumours series, 5th ed.):49–54.

[4] Allison DB, Wakely PE Jr, Siddiqui MT, Ali SZ. Nodular fasciitis: a frequent diagnostic pitfall on fine-needle aspiration. Cancer Cytopathol. 2017;125(1):20–9. 27525591 10.1002/cncy.21768

[5] Rani D, Gupta A. Cytological diagnosis and misdiagnosis of nodular fasciitis. J Cytol. 2019;36(4):196–9. 31741577 10.4103/JOC.JOC_112_18 PMC6844020

[6] Forcucci JA, Bruner ET, Smith MT. Benign soft tissue lesions that May mimic malignancy. Semin Diagn Pathol. 2016;33(1):50–9. 26490572 10.1053/j.semdp.2015.09.007

[7] Wang XL, De Schepper AMA, Vanhoenacker F, De Raeve H, Gielen J, Aparisi F, et al. Nodular fasciitis: correlation of MRI findings and histopathology. Skeletal Radiol. 2002;31(3):155–61. 11935200 10.1007/s00256-001-0462-z

[8] Coyle J, White LM, Dickson B, Ferguson O, Wunder J, Naraghi A. MRI characteristics of nodular fasciitis of the musculoskeletal system. Skeletal Radiol. 2013;42(7):975–82. 23624727 10.1007/s00256-013-1620-9

[9] Goldblum JR, Folpe AL, Weiss SW. Enzinger and weiss’s soft tissue tumors. 7th ed. Philadelphia: Elsevier Inc.; 2020. pp. 203–14.

[10] Lu L, Lao IW, Yu XL, Wang J. Nodular fasciitis: a retrospective study of 272 cases from China with clinicopathologic and radiologic correlation. Ann Diagn Pathol. 2015;19(3):180–5. 25890487 10.1016/j.anndiagpath.2015.03.013

[11] Dinauer PA, Brixey CJ, Moncur JT, Furg-Smith JC, Murphey MD. Pathologic and MR imaging features of benign fibrous soft-tissue tumors in adults. Radiographics. 2007;27:173–87. 17235006 10.1148/rg.271065065

[12] De Schepper AM, Ramon FA, Degryse HR. Magnetic resonance imaging of soft tissue tumors. J Belge Radiol. 1992;75(4):286–96. 1459930

[13] De Schepper AM, Ramon FA, Degryse HR. Statistical analysis of MRI parameters predicting malignancy in 141 soft tissue masses. Rofo. 1992;156(6):587–91. 1617181 10.1055/s-2008-1032948

[14] Chen CK, Wu HT, Chiou HJ, Wei CJ, Yen CH, Chang CY, et al. Differentiating benign and malignant soft tissue masses by magnetic resonance imaging: role of tissue component analysis. J Chin Med Assoc. 2009;72(4):194–201. 19372075 10.1016/S1726-4901(09)70053-X

[15] Sápi Z, Lippai Z, Papp G, Hegyi L, Sápi J, Dezső K, et al. Nodular fasciitis: a comprehensive, time-correlated investigation of 17 cases. Mod Pathol. 2021;34(12):2192–9. 34381187 10.1038/s41379-021-00883-x PMC8592838

[16] Frei S, de Lange EE, Fechner RE. Case report 690. Nodular fasciitis of the elbow. Skeletal Radiol. 1991;20:468–71. 1925684 10.1007/BF00191095

[17] Wu SY, Zhao J, Chen HY, Hu MM, Zheng YY, Min JK, et al. MR imaging features and a redefinition of the classification system for nodular fasciitis. Med (Baltim). 2020;99(45):e22906. 10.1097/MD.0000000000022906 PMC7647516 33157932

[18] Sukpanichyingyong S, Matsumoto S, Ae K, Tanizawa T, Hayakawa K, Funauchi Y, et al. Simultaneous bifocal and asymptomatic intramuscular nodular fasciitis of the thigh: a case report. Clin Case Rep. 2020;8(7):1213–6. 32695360 10.1002/ccr3.2872 PMC7364073

[19] Yanagisawa A, Okada H. Nodular fasciitis with degeneration and regression. Craniofac Surg. 2008;19(4):1167–70. 10.1097/SCS.0b013e318176ac1a 18650753

[20] Yu JS, Rofsky NM. Hepatic metastases: perilesional enhancement on dynamic MRI. AJR. Am J Roentgenol. 2006;186(4):1051–58. 16554578 10.2214/AJR.04.1698

[21] Weiss SW, Goldblum JR, Folpe A, editors. Enzinger and weiss’s soft tissue tumors. 7th ed. Philadelphia: Elsevier; 2019.

[22] Shimizu S, Hashimoto H, Enjoji M. Nodular fasciitis: an analysis of 250 patients. Pathology. 1984;16(2):161–6. 6462780 10.3109/00313028409059097

[23] Leung LYJ, Shu SJ, Chan ACL, Chan MK, Chan CHS. Nodular fasciitis: MRI appearance and literature review. Skeletal Radiol. 2002;31(1):9–13. 11807586 10.1007/s002560100411

[24] Matsuda I, Nakamura J, Ohkouchi M, Torii Y, Futani H, Tsukamoto Y, et al. Expression of p16 in nodular fasciitis: an implication for self-limited and inflammatory nature of the lesion. Int J Clin Exp Pathol. 2019;12(3):1029–34. 31933915 PMC6945175

[25] Kransdorf MJ, Murphey MD. Imaging of soft tissue tumors. 4th ed. Philadelphia: Lippincott Williams & Wilkins; 2013. pp. 232–7.

